# Streptococcal peptides and their roles in host-microbe interactions

**DOI:** 10.3389/fcimb.2023.1282622

**Published:** 2023-10-16

**Authors:** Emily R. Wahlenmayer, Daniel E. Hammers

**Affiliations:** Biology Department, Houghton University, Houghton, NY, United States

**Keywords:** *Streptococcus*, inflammation, peptides, Streptolysin S, quorum sensing, host-microbe interactions

## Abstract

The genus *Streptococcus* encompasses many bacterial species that are associated with hosts, ranging from asymptomatic colonizers and commensals to pathogens with a significant global health burden. Streptococci produce numerous factors that enable them to occupy their host-associated niches, many of which alter their host environment to the benefit of the bacteria. The ability to manipulate host immune systems to either evade detection and clearance or induce a hyperinflammatory state influences whether bacteria are able to survive and persist in a given environment, while also influencing the propensity of the bacteria to cause disease. Several bacterial factors that contribute to this inter-species interaction have been identified. Recently, small peptides have become increasingly appreciated as factors that contribute to Streptococcal relationships with their hosts. Peptides are utilized by streptococci to modulate their host environment in several ways, including by directly interacting with host factors to disrupt immune system function and signaling to other bacteria to control the expression of genes that contribute to immune modulation. In this review, we discuss the many contributions of Streptococcal peptides in terms of their ability to contribute to pathogenesis and disruption of host immunity. This discussion will highlight the importance of continuing to elucidate the functions of these Streptococcal peptides and pursuing the identification of new peptides that contribute to modulation of host environments. Developing a greater understanding of how bacteria interact with their hosts has the potential to enable the development of techniques to inhibit these peptides as therapeutic approaches against Streptococcal infections.

## 1 Introduction

The genus *Streptococcus* encompasses a diverse group of bacteria that occupy a variety of niches. Bacteria within this genus share several characteristics, including their identity as Gram-positive, facultatively aerobic cocci that ferment glucose and produce lactic acid as a by-product (Du Toit et al., 2014). Streptococci have traditionally been classified into Lancefield groups based on antigenic differences in their cell wall carbohydrates, and the taxonomy and nomenclature of the *Streptococcus* genus have changed significantly following the development of 16S rRNA sequencing (Facklam, 2002). Several Streptococcal species have clinical relevance as pathogens, and as many as 35 species of *Streptococcus* have been associated with invasive infections in humans (Krzyściak et al., 2014). Common species that are strongly associated with pathogenesis include *S. pyogenes* (Group A *Streptococcus*, GAS), *S. agalactiae* (Group B *Streptococcus*, GBS), *S. pneumoniae*, and *S. mutans* (Cole et al., 2008). Altogether, Streptococcal infections can range in severity from mild and self-limiting to severe and life-threatening, and continue to have a significant global health burden (Carapetis et al., 2005; O’Brien et al., 2009; Krzyściak et al., 2014; Pilarczyk-Zurek et al., 2022). Opportunistic infections and asymptomatic colonization of humans with these bacteria can also occur (Shaikh et al., 2010; Walker et al., 2014; Ndiaye et al., 2018; Armistead et al., 2019; Trimble et al., 2020). Furthermore, several Streptococcal species exist as normal parts of human and animal flora as commensals, enabling a wide variety of intermicrobial and host-microbe interactions (Du Toit et al., 2014; Baty et al., 2022).

As a result of the breadth of interactions between streptococci and their hosts, many streptococci must compete for space and resources in their host niche and interact either directly or indirectly with host immune systems (Kreth et al., 2005; Mehtälä et al., 2013; Wholey et al., 2019; Rostami et al., 2022). Depending on the context, streptococci are able to produce factors that promote either immune evasion or hyperinflammation beyond the immune response that is typical in response to infection. Host control over the type and extent of the immune response is critical for the effective clearance of pathogens, since insufficient bacterial clearance will result in disease progression, while excessive immune activation can damage the host (Castiglia et al., 2016). Several Streptococcal factors that enable interactions with the host immune system have been identified, especially in the context of pathogenesis. Many of these factors are proteinaceous, including the superantigens produced by GAS that act as mitogens for T cells (Proft and Fraser, 2007), the pore-forming toxin pneumolysin from *S. pneumoniae* (McNeela et al., 2010; Cole et al., 2021), and the β-hemolysin/cytolysin CylE from GBS (Doran et al., 2003). While many of the Streptococcal effectors that modulate host immunity are relatively large proteins, recent studies have increasingly identified small (<50 amino acid) peptides produced by species of *Streptococcus* as powerful molecules that have the ability to manipulate host immune systems. These peptides can induce these effects by directly stimulating the host, or indirectly as signaling peptides that primarily function by prompting intra-species changes in the expression of genes that regulate virulence, competence, biofilm formation, and other systems. Here, we review the recent studies that explore the functions of these Streptococcal peptides as they relate to disruption of host immune activity.

## 2 Streptolysin S

Pathogenic streptococci produce several virulence factors that enable them to cause disease. Among these virulence factors is the peptide toxin Streptolysin S (SLS). This peptide is most well-studied in the context of *S. pyogenes*, although the biosynthetic cluster that is responsible for the production of mature SLS is fairly well-conserved and distributed in several species of prokaryotes, including some that are distantly related to GAS (Lee et al., 2008; Molloy et al., 2014). Other streptococci can also produce SLS-like peptides, including *S. anginosus*, human isolates of *S. dysgalactiae* subsp. *equisimilius* (Group G *Streptococcus*, GGS), and animal pathogens including *S. suis*, *S. equi*, and *S. iniae* (Flanagan et al., 1998; Fuller et al., 2002; Humar et al., 2002; Tabata et al., 2013; Molloy et al., 2014; von Beek et al., 2019). Studies involving SLS have been complicated by the inability to successfully purify and elucidate the structure of mature SLS, as well as the non-immunogenic nature of the peptide. This is likely due in part to the extensive post-translational modifications that are introduced to the prepropeptide SagA, which produce a peptide with a molecular weight of approximately 2.7 kDa (Mitchell et al., 2009). Thiazole and oxazole heterocycles are introduced into the prepropeptide by a complex of the SagB, SagC, and SagD proteins, resulting in SLS being classified as a thiazole-oxazole modified microcin (TOMM) (Nizet et al., 2000; Datta et al., 2005; Lee et al., 2008). These modifications are critical for the bioactivity of the mature toxin, and similar modifications in TOMMs produced by other bacterial species are known to enable diverse biomolecular interactions (Roy et al., 1999; Mitchell et al., 2009). Previous studies have also indicated that the active form of SLS includes a complex made of the modified peptide and an RNA carrier (Bernheimer, 1967; Loridan and Alouf, 1986; Molloy et al., 2014). This indicates that the peptide portion of SLS may not act alone. However, combining the SagA prepropeptide with the SagB, SagC, and SagD modifying proteins results in bioactive SLS *in vitro*, suggesting that the oligonucleotide may not always be necessary for the activity of the toxin (Lee et al., 2008). SLS is most well-known for its ability to rapidly lyse erythrocytes, which was traditionally attributed to the ability of the toxin to disrupt host membranes (Duncan and Mason, 1976; Nizet et al., 2000; Carr et al., 2001). However, recent studies have indicated that SLS lyses host cells by targeting membrane proteins that are associated with ion transport (Higashi et al., 2016; Hammers et al., 2022), and several reports have expanded the role of SLS in the context of Streptococcal infections. The toxin is known to play an important role in invasive GAS infections (Betschel et al., 1998; Datta et al., 2005; Hirose et al., 2019), and is able to disrupt multiple host cells in addition to erythrocytes.

GAS infections have been associated with the induction of pro-inflammatory signaling cascades and programmed cell death (Tsai et al., 2006), and SLS has been increasingly recognized as a key Streptococcal factor that is responsible for driving damaging hyperinflammation and manipulating cell signaling in the host. SLS and Streptolysin O (SLO), another cytolysin produced by GAS (described in more detail below), were associated with the induction of inflammatory signaling and necrosis in macrophages (Goldmann et al., 2009). Here, a double mutant that was deficient for both SLS and SLO expression caused in reduced cytotoxicity in murine macrophages compared to the wild-type GAS strain, which corresponded with the reduced ability of the double mutant to recruit neutrophils to the site of infection. These results indicated that the streptolysins are able to trigger pro-inflammatory signals during infections (Goldmann et al., 2009). However, a more recent study using human macrophages suggested that SLS had no effect on the production of the pro-inflammatory cytokines IL-1β and TNF-α, nor the anti-inflammatory cytokines IL-10 and CXCL10 (Latvala et al., 2014). Regardless, SLS has since also been shown to be a strong pro-inflammatory effector in other contexts. For example, human keratinocytes experience a reduction in pro-survival Akt signaling and an upregulation of p38 MAPK signaling in response to SLS, followed by NF-κB activation and the increased expression of pro-inflammatory cytokines (Flaherty et al., 2015). This identified a potential link to the NF-κB activation that had previously been observed during GAS infections (Okahashi et al., 2003; Tsai et al., 2006). Subsequent studies demonstrated that IL-1β was especially upregulated in an SLS-dependent manner during these infections (Flaherty et al., 2018), providing additional support for the role of this cytokine in GAS infections (Wang et al., 2008; Valderrama et al., 2017; Richter et al., 2021a; Richter et al., 2021b). SLS appears to induce these signaling events in keratinocytes by targeting the membrane protein NBCn1 in this context (Hammers et al., 2022), thereby allowing GAS to promote disease severity by manipulating the host immune system and triggering excessive inflammation during infections. Exposure to SLS also triggers pro-inflammatory responses in host cells other than keratinocytes. For example, SLS from *S. anginosus* subsp. *anginosus* induces the expression of several genes in human oral squamous cell carcinoma (HSC-2) cells (Yamada et al., 2023). When these cells were incubated with supernatants from SLS-producing streptococci, the inflammatory cytokines IL-6 and CXCL8 were significantly upregulated, along with genes involved in the early growth response and genes in the Fos and Jun families. These changes were linked to the SLS-dependent influx of Ca^2+^ ions into the HSC-2 cells, further indicating a connection between SLS activity and ion flux (Yamada et al., 2023). In addition, SLS was identified (von Beek et al., 2019) as an important factor from *S. equi* that triggers p38 and Erk1/2 signaling in murine bone-marrow derived mast cells. Interestingly, sublytic concentrations of the detergent saponin triggered a similar response, indicating that SLS-induced membrane perturbation enables *S. equi* to stimulate an innate immune response in the host (von Beek et al., 2019). Altogether, these results demonstrate that multiple species of *Streptococcus* use the peptide toxin SLS to trigger inflammation in the host during infections.

In addition to the evidence that SLS is a pro-inflammatory factor, other studies have indicated that the peptide can contribute to the ability of the producing *Streptococcus* to evade the host immune system. For example, in an adult zebrafish model, infection with SLS-deficient GAS resulted in a significantly greater recruitment of neutrophils to the infection site compared to infection with wild-type GAS (Lin et al., 2009). Furthermore, infection of human keratinocytes with SLS-deficient GAS allowed for greater neutrophil recruitment than infection with wild-type GAS, which was attributed to SLS impairing the ability of the keratinocytes to produce chemotactic signals to recruit the neutrophils (Lin et al., 2009). SLS has also been shown to be required but not sufficient for inhibiting neutrophil recruitment in a mouse model of GAS infection, and the ability of SLS to prevent the infiltration of neutrophils correlated with the ability of GAS to disseminate during the infections (Feng et al., 2017). These results have suggested that streptococci have the potential to use SLS in a variety of ways during interactions with their hosts. Context, including the producing species and strain of *Streptococcus* and the location and temporal stage of infection, appears to be especially important for the effects of SLS on host immunity.

## 3 Streptococcal signaling peptides

The ability of bacteria to communicate with each other and trigger coordinated responses in a bacterial population has become increasingly well-known in recent years. This process, which is known as quorum sensing (QS), involves the production, secretion, and detection of chemical signals by a group of bacteria, and controls processes including virulence factor expression, competence, bioluminescence, production of antimicrobial substances, sporulation, and biofilm formation or dispersal (Rutherford and Bassler, 2012; Solano et al., 2014; Abisado et al., 2018; Mukherjee and Bassler, 2019; Azimi et al., 2020). Gram-positive bacteria, including members of the genus *Streptococcus*, use ribosomally-produced peptides that undergo post-translational modifications as signals called pheromones or autoinducing peptides (Jimenez and Federle, 2014; Galante et al., 2015; Shanker and Federle, 2017). These peptides can either be imported into bacterial cells and sensed by cytosolic transcriptional regulators, or can accumulate extracellularly before detection by membrane-bound histidine kinases that are members of two-component signaling systems (Dunny and Leonard, 1997; Rocha-Estrada et al., 2010). The cytosolic transcription factors that sense imported peptides comprise the large RRNPP protein superfamily, including Rap from *Bacillus subtilis*, Rgg from various species of *Streptococcus*, NprR and PlcR from *Bacillus cereus*, and PrgX from *Enterococcus faecalis* (Rocha-Estrada et al., 2010; Do and Kumaraswami, 2016; Neiditch et al., 2017). Signaling by QS peptides typically relies on a positive feedback loop, and often involves additional inputs to the system to turn off QS-regulated genes when their expression is no longer beneficial to the bacteria. In streptococci, this is often accomplished by using endopeptidases to degrade peptide pheromones (Wilkening et al., 2016; Knoops et al., 2022b; Hu et al., 2023). The wide range of roles of QS systems, and particularly the ability to regulate the expression of virulence factors, underscores the importance of QS for allowing streptococci to manipulate their environment. Here, we review Streptococcal signaling peptides and systems that have been associated with the production of bacterial factors that contribute to virulence or modulate host immunity.

### 3.1 Regulator gene of glucosyltransferase family

**Figure 1:**
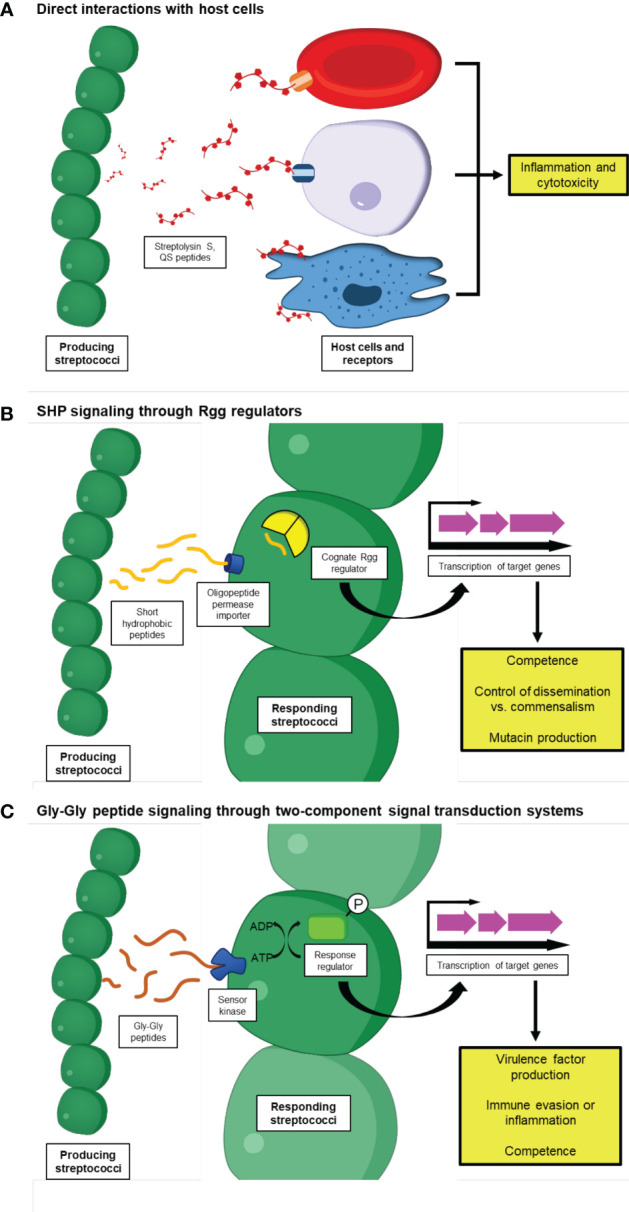
Streptococcal peptides as modulators of host-microbe interactions. Species from the genus *Streptococcus* produce a diverse array of peptides that enable a variety of interactions with their hosts. **(A)** These peptides sometimes interact directly with host cells to induce their effects, such as Streptolysin S, which triggers the lysis of several host cell types including epithelial cells, erythrocytes, and macrophages. SLS is also associated with the induction of pro-inflammatory signaling cascades and programmed cell death. Some host cells can also directly sense Streptococcal peptides primarily used as signaling molecules and subsequently produce immune responses, such as mast cells which use orthologs of the Mrgprb2 receptor to trigger inflammatory signaling upon detection of Streptococcal competence-stimulating peptide (CSP). **(B, C)** Streptococcal peptides can also modulate the immune systems of their host and influence bacterial virulence indirectly, by acting as pheromones to induce or repress the transcription of other genes associated with host-microbe interactions. In streptococci, these can be further subdivided into two categories, including: **(B)** Peptides that interact with RRNPP superfamily transcriptional regulators, including short hydrophobic peptides (SHPs) which interact with regulator gene of glucosyltransferase proteins upon importation into the receiving cell by oligopeptide permease proteins, and **(C)** Peptides that contain a double-glycine motif and rely on two-component signal transduction systems involving a sensor kinase and response regulator to alter transcription. These signaling peptides are associated with a variety of processes, including competence, colonization, and the production of other Streptococcal virulence factors.

Many streptococci possess the ability to produce a class of peptides known as short hydrophobic peptides (SHPs). These peptides are synthesized as precursor polypeptides which mature into active pheromones as a part of their export process from the producing streptococci (Aggarwal et al., 2014; Chang and Federle, 2016). These SHPs then accumulate in the extracellular space, and are eventually taken up by oligopeptide transporters in other nearby streptococci, where they bind to transcription factors in the regulator gene of glucosyltransferase (Rgg) family (Chang et al., 2011; Cook and Federle, 2014; Jimenez and Federle, 2014). This family was named for the discovery of its initial member in *Streptococcus gordonii* (Sulavik et al., 1992; Sulavik and Clewell, 1996), but subsequent studies have identified a wide variety of Rgg genes that are distributed across the genus *Streptococcus*, which are frequently located near their corresponding SHP gene (Ibrahim et al., 2007; Fleuchot et al., 2011). Several of these SHP-Rgg pairs have been shown to regulate Streptococcal factors that enable the bacteria to interact with and respond to their hosts (**Figure 1B**).

#### 3.1.1 Rgg/RopB

Different species of *Streptococcus* utilize a variety of SHP-Rgg systems to control gene expression. In *S. pyogenes*, four different Rgg proteins have been identified (Jimenez and Federle, 2014). One such protein is the regulator of proteinase B (RopB, also called Rgg). Although RopB was classified as an Rgg protein based on homology, it was initially referred to as a stand-alone regulator as a result of its cognate signal peptide being unknown because of the absence of an identifiable coding region for a potential SHP (Kreikemeyer et al., 2003; Fleuchot et al., 2011). However, a recent study identified a secreted peptide that is translated in a mature, leaderless form which binds RopB and modulates its DNA-binding activity. This peptide was named SpeB-inducing peptide (SIP) as a result of its ability to control the expression of the cysteine protease SpeB. This study suggested that at high cell densities, GAS produces SIP, triggering the RopB-dependent expression of the *speB* gene (Do et al., 2017). These findings provided additional support for the relationship between RopB and SpeB expression, which had been previously described (Neely et al., 2003; Makthal et al., 2016). SpeB has been extensively studied as a GAS virulence factor, and has received attention as a protease that helps GAS evade host immune systems. This is largely due to the wide range of proteinaceous substrates that are cleaved by SpeB, including the immunoglobulins IgA, IgD, IgE, IgM, and IgG, enabling bacterial survival in human blood (Collin and Olsén, 2001; Collin et al., 2002; Eriksson and Norgren, 2003). SpeB can also cleave the opsonizing complement component C3b, providing another example of how GAS uses SpeB to disrupt host immunity (Terao et al., 2008). However, a more recent study suggested that SpeB-mediated degradation of immunoglobulins does not occur under physiological conditions (Persson et al., 2013), indicating that the relationship between SpeB and immune evasion is complicated.

Although RopB is most commonly associated with SpeB, its regulon usually includes several other genes. In fact, the number of genes regulated by RopB appears to be dependent on the particular GAS strain being studied and the bacterial growth context, ranging from 0.15 percent of the genome of MGAS5005 to 31 percent of the genome of NZ131 (Dmitriev et al., 2006; Dmitriev et al., 2008; Hollands et al., 2008; Carroll et al., 2011; Do et al., 2017). Several of the genes that have been linked to RopB regulation have important roles in virulence and disruption of the host immune system. For example, transcripts of the *sagA* gene that encodes the precursor peptide of SLS were increased in an RopB mutant strain. This increase was attributed to reduced transcription of the *csrRS* regulator in this background (Chaussee et al., 2002). As described previously, SLS plays a major role in the ability of GAS to disrupt host immunity, indicating that peptide pheromone-mediated bacterial signaling can have additional effects on the interactions between streptococci and their hosts. Other factors that have been associated with RopB regulation include SLO and C5a peptidase (Chaussee et al., 2002). SLO is a pore-forming toxin that has been shown to activate IL-1β signaling and induce a form of programmed cell death called pyroptosis in macrophages through the NLRP3 inflammasome (Harder et al., 2009; Timmer et al., 2009; Keyel et al., 2013; Vande Walle and Lamkanfi, 2016; Valderrama and Nizet, 2018). C5a peptidase (also called SCPA) disrupts the complement component of the immune system by cleaving the host chemotactic peptide C5a, which is involved in binding polymorphonuclear leukocytes (Cleary et al., 1992). In addition, a more recent study showed that C5a peptidase was also able to cleave the complement components C3 and C3a (Lynskey et al., 2017) Cleavage of these complement components reduced the efficacy of the complement system, as evidenced by a reduced opsonization of GAS by C3 and reduced the ability of neutrophils to clear the bacteria. Furthermore, C5a peptidase also promoted the dissemination and virulence of GAS in an *in vivo* model with mice lacking both C3 and C5. This suggested that C5a peptidase has broad proteolytic activity and can influence GAS pathogenesis in both complement-dependent and complement-independent ways (Lynskey et al., 2017). Altogether, this indicates that the transcriptional regulator RopB, which acts in a QS circuit with a Streptococcal signaling peptide, impacts the transcription of several GAS factors that modulate host immunity.

#### 3.1.2 Rgg2/3

Several other Rgg transcriptional regulators are also present in streptococci. Among these regulators are Rgg2 and Rgg3 in GAS, which respond to the peptide pheromones SHP2 and SHP3. The transcriptional regulators share a high degree of similarity, as do the SHP2 and SHP3 peptides, which are functionally similar in GAS and have been referred to collectively as SHP (Chang et al., 2011; Aggarwal et al., 2014; Jimenez and Federle, 2014; Rahbari et al., 2021). However, despite the fact that both peptides are capable of activating the Rgg2/3 QS circuit, there is evidence that they have some differences in the degree to which they activate the circuit (Chang et al., 2011; LaSarre et al., 2013). Mature and active peptide pheromones can vary in length, with different lengths corresponding to different binding affinities to Rgg proteins, possibly indicating additional levels of transcriptional control by this system (Aggarwal et al., 2014). Overall, Rgg3 acts as a transcriptional repressor until SHP concentrations reach a sufficient level for its inactivation, while SHP concurrently activates the transcriptional activator Rgg2, allowing for transcription of the target genes (Chang et al., 2011). Orthologous regulators have been found in other species of streptococci, although *S. pyogenes* appears to be the only species that encodes both Rgg2 and Rgg3 with their corresponding peptides (Cook et al., 2013). The transcriptional regulator RovS and its corresponding pheromone SHP1520 act as an Rgg2/SHP2-like system in *S. agalactiae* (Group B *Streptococcus*, GBS), and strains of *S. dysgalactiae* subsp. *equisimilus* also contain an orthologous system. In addition, orthologs of Rgg3/SHP3 have been found in *S. porcinus, S. pnuemoniae*, and *S. thermophilus* (Cook et al., 2013). Rgg regulators of some streptococci are able to respond to SHPs from other Streptococcal species, indicating that cross-talk between these systems is possible (Cook et al., 2013; Fleuchot et al., 2013).

Streptococci use Rgg2/3-like systems to regulate the expression of a variety of genes. In GAS, quorum sensing through the Rgg2/3 system has been associated with biofilm formation and lysozyme resistance (Chang et al., 2011; Gogos et al., 2018). More recently, this system was shown to enable GAS to suppress innate immunity of the host (Rahbari et al., 2021). In this study, disruption of the QS circuit triggered NF-κB activation and subsequent production of TNF-α and IL-6 in macrophages. Similar results were obtained across multiple GAS strains (NZ131, M1T1 5448, and HSC5) and multiple host cell lines, including human-derived differentiated THP-1 monocytes. However, this was not universal among all GAS strains tested, since macrophages infected with MGAS315 experienced minimal TNF-α induction regardless of the status of the QS system (Rahbari et al., 2021). The immunosuppressive effects of this system were linked to a portion of the regulon of Rgg2/3 (*spy49_0450-0460*), which encodes a putative biosynthetic gene cluster, and not the bacterial protein StcA, which mediates biofilm production and lysozyme resistance and is also regulated by Rgg2/3 QS (Chang et al., 2011; Rahbari et al., 2021).

In GBS, the Rgg2-like RovS/SHP1520 system regulates the expression of several factors, including *fbsA*, gbs0230, *sodA*, and the *cyl* operon (Samen et al., 2006; Pérez-Pascual et al., 2015). Expression of the *fbsA* gene is negatively regulated by the RovS/SHP1520 system (Samen et al., 2006), although it has been suggested that this regulation may be indirect (Pérez-Pascual et al., 2015). In contrast, gbs0230, *sodA*, and the *cyl* operon appear to be positively regulated by the RovS/SHP1520 system, because a *rovS* mutant exhibited reduced transcription levels of these genes (Samen et al., 2006). Each of these factors appears to play a role in either virulence or peptide signaling. The protein encoded by the *fbsA* gene is a bacterial receptor for fibrinogen, and has been associated with adherence to host epithelial cells (Schubert et al., 2004). In addition, peptide fragments of FbsA are immunogenic and have been studied as potential vaccine antigens (Papasergi et al., 2013). The protein encoded by gbs0230 has been reported as an Rgg-like paralog, indicating that this system could potentially be involved in the regulation of other Transcriptional regulation systems (Glaser et al., 2002; Samen et al., 2006). GBS uses a superoxide dismutase encoded by the *sodA* gene to mitigate the effects of reactive oxygen species, helping to protect the bacteria from macrophage-mediated cell death and evade the host immune system (Poyart et al., 2001).

The most well-studied of these genes regulated by the RovS/SHP1520 system are those that are found in the *cyl* operon that is responsible for the production of the GBS β-hemolysin/cytolysin (β-H/C, CylE), which has several roles in the context of infection (Pritzlaff et al., 2001; Rajagopal, 2009). This virulence factor is associated with increased inflammation during GBS-mediated arthritis, and is significantly upregulated in GBS prosthetic joint infections (Puliti et al., 2000; Cho et al., 2021). The increase in disease severity caused by hyperhemolytic mutants in an arthritis model correlated with an increase in both local and systemic levels of the pro-inflammatory cytokine IL-6 (Puliti et al., 2000). A subsequent study showed that hemolytic bacteria that produce β-H/C induce higher levels of TNF-α in human monocytes compared to a non-hemolytic strain, and that this increase in TNF-α production correlated with reduced intracellular bacterial survival within the monocytes (Sagar et al., 2013). Furthermore, β-H/C-producing GBS induced a more pronounced pro-inflammatory response compared to a β-H/C-deficient mutant in bladder epithelial cells, including increased production of the cytokines IL-6, IL-8, and IL-1α (Kulkarni et al., 2013). Similar inflammatory responses were observed in an *in-vivo* urinary tract infection model, although β-H/C expression did not give any apparent selective advantage to the wild-type strain in this model (Kulkarni et al., 2013). Altogether, these studies indicate that the Rgg2 paralog RovS and its cognate peptide pheromone SHP1520 play a major role in regulating a variety of GBS factors that allow the bacteria to interact with the host immune system.

#### 3.1.3 Rgg4 (ComR)

Quorum sensing systems are also used by streptococci to regulate the expression of genes that enable genetic competence. In some Streptococcal species, the transcriptional regulator ComR is directly involved in this process (Fontaine et al., 2010; Mashburn-Warren et al., 2010). ComR is an Rgg-type regulator and can be found in GAS, where it is also called Rgg4 (Chang et al., 2011), as well as in the Salivarius, Bovis, and Mutans groups of streptococci and some Viridans streptococci (Fontaine et al., 2010; Mashburn-Warren et al., 2010). The corresponding peptide pheromone for this system is encoded by the *comS* gene, and the mature peptide is called XIP, for *sigX*-inducing peptide (Mashburn-Warren et al., 2010). Binding of XIP to ComR induces dimerization of the transcriptional regulator and enhances additional transcription of *comS* through a positive feedback loop, resulting in amplification of the early competence stage (Talagas et al., 2016; Ledesma-Garcia et al., 2020; Knoops et al., 2022a). The SigX protein (also called ComX) is an alternative sigma factor that acts as a master regulator of late competence genes (Lee and Morrison, 1999; Mashburn-Warren et al., 2012). The ability of ComRS to control both early and late competence has been an active area of research, and it was recently discovered that the well-described two-component regulator system CovRS controls the amount of ComR in *S. salivarius*, thereby enabling this bimodal regulation (Knoops et al., 2022a). SigX can be found throughout the genus *Streptococcus*, and the groups in which this factor is controlled by the ComRS system can be further subdivided into two classes based on features of the QS system, with the most important differentiator being the C-terminal region of XIP (Claverys and Martin, 2003; Fontaine et al., 2010; Mashburn-Warren et al., 2010; Fontaine et al., 2013; Cook and Federle, 2014).

Intracellular signaling by XIP has also been observed in *S. mutans*, indicating that the peptide can contribute to competence control without needing to be exported or accumulate extracellularly (Underhill et al., 2018). Importantly, competence in *S. mutans* appears to be under the control of an additional regulatory circuit called ComCDE (described below), indicating that competence is controlled by multiple inputs in this species (Son et al., 2012). Other peptides that are involved in ComRS-mediated competence in *S. mutans* have been identified, including XrpA (ComX regulatory peptide A), which is encoded within the coding sequence of the *comX* gene. An XrpA-deficient mutant exhibited increased expression of both *comX* and the peptide pheromone precursor *comS*, demonstrating that this peptide negatively regulates competence in *S. mutans* (Kaspar et al., 2015; Kaspar et al., 2018). Additionally, XrpA has been associated with an additional operon involved in competence regulation termed r*crRPQ* (for Rel and Competence Related) (Seaton 2015). If XrpA was inactivated without altering the primary sequence of the ComX protein that it was encoded within, the production of ComX could be restored in a strain with a nonpolar mutation of *rcrR*, matching the phenotype that was observed in a strain with a polar mutation of *rcrR* (Kaspar et al., 2015). Studies involving how exactly the *rcrRPQ* operon contribute to the control of competence in *S. mutans* are ongoing (Kaspar and Walker, 2019). Regardless, it is clear that competence in streptococci is a highly controlled process, and peptides play a major role in this control.

ComR-mediated competence in GAS has been linked to biofilm development, since a ComR-deficient strain of MGAS315 was impaired in its ability to form biofilms (Marks et al., 2014). Furthermore, this study indicated that biofilm-associated GAS is naturally competent, demonstrating natural competence in pyogenic streptococci for the first time and linking ComR-XIP signaling with biofilm formation (Marks et al., 2014). As described below, altering the bacterial surface and forming biofilms represents an avenue by which streptococci are able to interact with immune systems of their hosts. Furthermore, competence has been associated with virulence through releasing or exposing virulence factors (Lin et al., 2016; Minhas et al., 2023), which could further enable streptococci to modulate host immunity during infections.

#### 3.1.4 Other Rgg circuits

As more genome sequences for bacterial strains become available, additional putative Rgg-like QS circuits are being identified. For example, Rgg/SHP systems have been associated with the expression of biosynthetic operons that include radical-SAM (*S*-adenosyl-L-methionine) (RaS) enzymes, which in turn produce a diverse family of peptides known as RaS-ribosomally synthesized and post-translationally modified peptides (RaS-RiPPs). These systems are found in many streptococci, although many of the resulting natural products are either undiscovered or have unclear functions (Clark et al., 2022). However, some Streptococcal RaS-RiPPs have been shown to act as antimicrobial peptides, possibly to enable some streptococci to outcompete related bacteria that are attempting to occupy a similar niche. Some examples of these include Streptosactin from *S. thermophilus* and tryglysin from *S. mutans* (Bushin et al., 2020; Rued et al., 2021; Clark et al., 2022). The continued discovery and characterization of RaS-RiPPs will likely identify several new peptides with important roles in streptococci-host interactions. In addition, seven putative Rgg systems have been identified in *S. pneumoniae*, and their distribution varies depending on the strain (Zhi et al., 2018). One study used the well-characterized D39 strain and identified five such systems. These included the Rgg144 regulator and its cognate pheromone SHP144, which was found in all of the 31 evaluated pneumococcal strains and was common in the related species *S. pseudopneumoniae*, *S. mitis*, and *S. oralis*. Zhi and colleagues characterized this core system and the accessory Rgg system Rgg939/SHP939, which is less common in pneumococcal genomes (Junges et al., 2017; Zhi et al., 2018). The predicted binding site of the Rgg939 protein in the promoter region of its SHP is similar to the promoter region recognized by Rgg2/3 in GAS, and SHP3 in GAS is identical to *S. pneumoniae* SHP939 in terms of its amino acid sequence, indicating some similarities between these species. Another Rgg system, consisting of Rgg1518 and its cognate peptide pheromone SHP1518, was identified as an accessory system and was also characterized recently (Zhi et al., 2018; Shlla et al., 2021). Each of these Rgg systems is implicated in pneumococcal virulence, since mutant strains defective in these systems were shown to be less able to colonize and cause disease in mouse models of pneumococcal infection (Zhi et al., 2018; Shlla et al., 2021). However, another recent study indicated that a mutant deficient in the Rgg939 system was not significantly affected in terms of virulence, and instead showed that overexpression of the Rgg939 regulon resulted in impaired fitness in an infection model (Junges et al., 2017). Zhi and colleagues attributed these differences to the time points at which samples were collected in these studies, indicating that a longer infection time may be necessary to observe a significant impact of Rgg939 on pneumococcal virulence (Zhi et al., 2018).

The regulons of these pneumococcal Rgg systems are diverse and often variable, depending on environmental conditions. Rgg144 has a particularly large regulon, including genes involved in capsule biosynthesis and another regulatory gene encoding a Gly-Gly peptide named virulence peptide 1 (VP1) (Cuevas et al., 2017; Zhi et al., 2018). VP1 is found in the locus that is most highly upregulated by Rgg144 and is a major contributor to biofilm formation and dissemination during pneumococcal infection (Cuevas et al., 2017). This locus is also regulated by Rgg939 in a sugar-dependent manner, being upregulated when pneumococci are grown with mannose as a carbon source, and downregulated when they are grown with galactose (Zhi et al., 2018). The ability of Rgg939 to also regulate a locus involved in biofilm formation supports previous results that indicate that overexpression of this system reduces biofilm formation, while pneumococcal CFUs in biofilms are significantly increased when Rgg939 is deleted (Junges et al., 2017). The Rgg1518 system works to repress capsule biosynthesis while also playing a role in other metabolic pathways (Shlla et al., 2021).

Changes in the bacterial surface and propensity to form and maintain biofilms have important implications for how pneumococci interact with the host immune system, indicating that the peptide pheromones that contribute to the regulation of these cellular processes are important for enabling the bacteria to survive in the host environment. Biofilm-associated pneumococci have traditionally been thought of as avirulent, largely due to the observation that mutants that lack the ability to produce capsular polysaccharide (CPS) form highly robust biofilms and are unable to prevent opsonophagcytosis (Moscoso et al., 2006; Hyams et al., 2010; Gilley and Orihuela, 2014). Furthermore, biofilm dispersal has been associated with an increase in the expression of virulence genes and dissemination (Marks et al., 2013). However, biofilm-associated pneumococci are able to avoid complement-mediated immunity by preventing the binding of C3b, C-reactive protein, and C1q to the bacterial surface (Domenech et al., 2013). Pneumococci in biofilms were also recently shown to lyse macrophages in a pneumolysin-dependent manner, identifying a mechanism of active immune suppression when the bacteria are in a biofilm state (Shenoy et al., 2017). Controlling biofilm formation through Rgg/SHP systems can therefore provide *S. pneumoniae* with a way to disrupt the host immune system during colonization and infection.

### 3.2 Tpr/Phr systems

*Streptococcus pneumoniae* utilizes an additional QS system involving Phr peptides and Tpr (transcription factor regulated by Phr peptide) regulators, which are homologs of the PlcR regulators in the RRNPP superfamily (Rocha-Estrada et al., 2010; Hoover et al., 2015; Aggarwalid et al., 2020). Two Phr peptides (PhrA and PhrA2) have been experimentally characterized in *S. pneumoniae*, while at least two other Phr peptides (PhrB and PhrC) have been identified (Hoover et al., 2015; Kadam et al., 2017). The TprA2/PhrA2 system is especially common in the PMEN1 lineage of pneumococcus, which is known for its propensity for antibiotic resistance and switching serotypes, enabling the development of vaccine escape strains (Reinert et al., 2005; Croucher et al., 2011; Kadam et al., 2017). However, the association of PMEN1 strains with invasive pneumococcal disease appears to be more attributable to its high rates of carriage rather than its propensity for virulence (Sjöström et al., 2006). Tpr regulators typically act as transcriptional repressors, such that binding of the cognate Phr peptide activates the expression of the regulated genes (Hoover et al., 2015; Aggarwalid et al., 2020). Some crosstalk between these systems is possible, since PhrA2 is able to activate TprA/PhrA signaling in both PMEN1 and D39 strains of *S. pneumoniae*, but PhrA is unable to activate TprA2/PhrA2 signaling (Kadam et al., 2017). Furthermore, TprA/PhrA signaling is dependent on the carbon source that is available to the bacteria, and the *tprA* gene is influenced by the positive regulator GlnR and the negative regulator CcpA in different conditions (Motib et al., 2019). The dependence of TprA activation on the presence of galactose or mannose suggests that this system is likely active in the host nasopharynx, due to the presence of these molecules in host glycans (King et al., 2006). The influence of available carbon sources on these systems indicates that these QS circuits are tightly regulated and are able to respond to environmental conditions by controlling gene expression.

TprA/PhrA systems can regulate a variety of genes, including some involved in producing lanthionine-containing peptides, controlling sugar utilization, and neuraminidase production (Hoover et al., 2015; Kadam et al., 2017; Motib et al., 2017; Motib et al., 2019). The TprA/PhrA circuit has been shown to be essential for pneumococcal virulence in a chinchilla otitis media model using the D39 strain, and both TprA- and PhrA- deficient bacteria were less able to survive and disseminate in mouse models of pneumococcal infection (Motib et al., 2017; Motib et al., 2019). Mechanistic reasons for the attenuation of these mutants in these models is lacking, but Motib and colleagues speculate that this is related to the inability of these mutants to process and transport host sugars *in vivo* (Motib et al., 2019). In contrast, the TprA2/PhrA2 system appears to promote commensalism rather than disease progression, since a strain deficient in TprA2 induced higher mortality in a murine model than the corresponding wild-type strain with the TprA2 system intact (Kadam et al., 2017). This result was linked to the ability of TprA2 to negatively regulate the *lcpAMT* genes involved in the synthesis of a lanthionine-containing peptide, LcpA. A deletion of *lcpAMT* in the TprA2-knockout background eliminated the ability of the TprA2 mutant to cause increased mortality, identifying LcpA as a virulence determinant. The exact function of LcpA remains unknown, despite its appearance as a bacteriocin with antimicrobial activity based on its sequence (Kadam et al., 2017). Additional studies to elucidate the function of LcpA would have implications for understanding how pneumococcal strains are able to switch from commensal to pathogenic states. Being able to alter the viability of bacteria in different *in vivo* conditions opens the possibility that this peptide has immunomodulatory properties. Regardless, it is clear that TprA/PhrA-like systems play an important role in pneumococcal communication and pathogenesis.

### 3.3 Double glycine peptide signaling systems

In addition to peptides that are imported into bacterial cells and bind cytosolic transcription factors to induce their effects, several streptococci use members of the double glycine family of peptides to carry out intercellular signaling and control the transcription of genes. These peptides are named for their Gly-Gly motif that is found in their conserved leader sequences (Håvarstein et al., 1994). They are translated as propeptides that are processed into their mature forms by an accessory domain on the transporter responsible for export of the peptides from the bacterial cell (Håvarstein et al., 1995a). When these peptides are used as signaling molecules, they are sensed by two-component signal transduction systems on the receiving cell, which propagate the signal inside the cell to induce a response (**Figure 1C**) (Cook and Federle, 2014). Peptides in this family that are produced by *Streptococcus* species and have been linked to manipulation of host immunity will be discussed here.

#### 3.3.1 ComCDE

As previously described, several species of *Streptococcus* are able to regulate the expression of genes involved in competence through the Rgg-type QS system ComRS. However, many streptococci that do not utilize this system are able to regulate competence through the use of a different QS system called ComCDE. Specifically, this system is found in the Mitis and Anginosus groups of *Streptococcus* in addition to *S. mutans*, which also utilizes the ComRS system to influence competence (Cook et al., 2013; Shanker and Federle, 2017; Kaspar and Walker, 2019). It includes the ABC transporter with processing and export functions ComAB, the precursor to the peptide pheromone ComC, and the members of the two-component signal transduction system ComDE (Håvarstein et al., 1994; Håvarstein et al., 1995a; Håvarstein et al., 1995b; Pestova et al., 1996). ComC is produced with a double glycine motif, and during the export process, it is cleaved by ComAB to produce the active peptide pheromone CSP (competence-stimulating peptide) (Håvarstein et al., 1995a). As CSP accumulates in the extracellular space, it binds to the ComD histidine kinase receptor on receiving cells, leading to the transfer of a phosphate group to the cytosolic response element ComE, which then activates the transcription of target genes (Lee and Morrison, 1999; Martin et al., 2013). Phosphorylated ComE recognizes conserved promoter site called a ComE-binding site (Ceb) to control transcription, and this element is found upstream of the *comAB* and *comCDE* operons, meaning that CSP-mediated signaling can further activate the ComCDE system (Ween et al., 1999). In addition, recent evidence indicates that the transcription of *comCDE* in *S. pneumoniae* is influenced by catabolite control protein A (CcpA), indicating that available carbon sources can also contribute to signaling through this system (Zhang et al., 2023).

The ComCDE system regulates a variety of genes, several of which are associated with controlling Streptococcal interactions with their hosts. The system is most well-known for stimulating competence by inducing transcription of the *comX* gene, leading to the production of the SigX alternative sigma factor that was described above. ComCDE-mediated *comX* expression has a complicated relationship with pathogenesis. For example, the addition of exogenous CSP to a mutant strain of *S. sanguinis* that was unable to produce its own pheromone induced competence in the strain, but it was later found that neither *comX* nor the *comCDE* operon were required for virulence in a rabbit model of infective endocarditis, despite eliminating competence in these strains (Callahan et al., 2011; Rodriguez et al., 2011). In another study, deletion of the response regulator ComE in *S. pneumoniae* resulted in bacteria that were able to more effectively colonize the upper respiratory tract of an infant rat model than their wild-type counterparts. Deleting ComD had similar effects if the mutation was made in such a way that expression of ComE was reduced, but colonization was impaired if the mutation resulted in an increase in *comE* transcription instead (Kowalko and Sebert, 2008). This result could explain an apparent discrepancy with a previous study, which indicated that ComCDE was important for virulence in pneumococcus because deletion of *comD* significantly impaired virulence in terms of bacterial burden in murine models for both respiratory tract and systemic infections (Bartilson et al., 2001).

Later studies demonstrated that in addition to *comX*, ComCDE signaling can have other roles that are related to Streptococcal virulence. For example, ComE has been shown to positively regulate the *briC* gene in *S. pneumoniae.* This gene encodes the Gly-Gly BriC (biofilm-regulating peptide induced by competence) peptide, which is named for its strong contribution to late-stage biofilm formation and is well-conserved across strains of *S. pneumoniae*. Furthermore, this peptide appears to be secreted by the ComAB transporter, indicating that biofilm formation and this competence pathway are closely linked in pneumococcus (Aggarwal et al., 2018). The *briC* gene is cotranscribed with the *fab* (fatty acid biosynthesis) gene cluster FASII in pneumococcus, and disrupting the peptide alters bacterial membrane properties by reducing the production of unsaturated fatty acids (Aggarwal et al., 2021). As described above, altering the cell surface composition and ability to form biofilms has important implications for host-pathogen interactions, and disruption of the *briC* gene or the ComE-binding box within the *briC* promoter resulted in a reduced ability to colonize the nasopharynx of mice (Aggarwal et al., 2018). These results demonstrate that interbacterial sensing through the peptide pheromone CSP can influence how pneumococci interact with their hosts.

Recent evidence also indicates that host cells are able to directly sense CSP from *Streptococcus* species and trigger a corresponding immune response. In one study, mice infected with wild-type D39 *S. pneumoniae* exhibited a greater recruitment of mast cells to the site of infection and higher levels of TNF-α in the nasal lavage fluid compared to mice that were deficient in ComC (Pundir et al., 2019). These results were associated with the Mrgprb2 protein, a G-protein coupled receptor (GPCR) that has been shown to sense inflammatory peptides and contribute to pseudo-allergic drug reactions (McNeil et al., 2015). Mice lacking this receptor were less effective at clearing bacteria during infection, and activation of Mrgprb2 with exogenous CSP-1 during infection enhanced bacterial clearance. The authors also demonstrated that MRGPRX2, the human ortholog of this receptor, was able to respond to CSP (Pundir et al., 2019). CSP-1 from *S. mutans* can also stimulate the production of CXCL8/IL-8, TNF-α, and IL-6 in gingival epithelial cells. This response was mediated by the bitter taste receptor T2R14, another GPCR (Medapati et al., 2021). These results demonstrate that the host has the potential to “listen in” on bacterial communication signals and respond, identifying a potential mechanism by which the innate immune system can recognize Gram-positive bacteria.

The relationship between ComCDE signaling, competence, and virulence is more complicated in *S. mutans*. While some evidence indicates that both ComCDE and ComRS contribute to competence in this species depending on media composition (Son et al., 2012), other evidence suggests that CSP-mediated signaling regulates bacteriocin production, but not competence, in this context (Reck et al., 2015). In addition, the ComCDE system appears to be related to the *rcrRPQ* operon that was introduced above. Two open reading frames encoding short peptides were found near the 3’ end of the *rcrQ* transcript. In the mutant strain with a nonpolar deletion of *rcrR* that was non-transformable, deletion of both peptide-encoding open reading frames restored bacterial sensitivity to CSP and resulting competence. The specific functions of the peptides remain unclear, although it was suggested that one or both peptides acted as negative regulators of competence (Ahn et al., 2014; Kaspar and Walker, 2019). Regardless, these studies demonstrate the importance of several peptides on both the induction and regulation of competence in *S. mutans*, which are reviewed further elsewhere (Hagen and Son, 2018; Kaspar and Walker, 2019).

#### 3.3.2 The Streptococcal invasion locus

The *sil* (Streptococcal invasion locus) is a group of genes found in a QS-regulated locus that is associated with Streptococcal virulence. The locus is most closely associated with GAS, although only about 25 percent of GAS strains have been shown to contain the locus, and most GAS strains that do not possess intact Sil retain fragments of the genomic region. In contrast, most GGS strains that have been sequenced contain the locus (Belotserkovsky et al., 2009; Michael-Gayego et al., 2013). The locus is also found in the Anginosus group of streptococci, where it is associated with a high amount of genetic variability and interspecies bacterial competition due to the cluster of bacteriocins often located near and regulated by the locus (Mendonca et al., 2017). GAS can also utilize *sil* to control the expression of bacteriocins, presumably to allow GAS to outcompete other bacteria during an infection (Hertzog et al., 2018). The *sil* locus was first identified using a transposon mutagenesis screen in a mouse model of necrotizing fasciitis, where it was found to contain a two-component signal transduction system (encoded by *silAB*), an ABC transporter (encoded by *silDE*), and the propeptide pheromone (encoded by *silCR*) (Hidalgo-Grass et al., 2002). It is noteworthy that the propeptide gene itself is transcribed from an open reading frame that overlaps the majority of the *silC* gene on the complementary strand that runs in the opposite direction, encoding a peptide with a Gly-Gly processing motif (Hidalgo-Grass et al., 2002; Hidalgo-Grass et al., 2004; Eran et al., 2007). Signaling through this system involves the mature SilCR peptide binding the SilB receptor, which then initiates the transfer of a phosphate group to the response regulator SilA, which goes on to stimulate the expression of other downstream genes (Eran et al., 2007; Belotserkovsky et al., 2009).

The Sil system has long been associated with Streptococcal pathogenesis, although more recent studies have found that the actual activity of the system may be dependent on the strain and infection context. Disruptions in the locus have previously been associated with losses in virulence and the ability of the bacteria to disseminate (Hidalgo-Grass et al., 2002; Kizy and Neely, 2009). However, strains used in these studies had nonfunctional start codons in the *silCR* gene, indicating that these strains were able to respond to exogenous pheromone if it were added, but were not able to produce their own. These mutations have been observed in multiple GAS strains that retain the Sil locus (Michael-Gayego et al., 2013). Additional support for the role of SilCR in Streptococcal pathogenesis was provided by Salim and colleagues. They used the MGAS166 strain (serotype M1) in a murine infection model and observed that the addition of SilCR had no impact on expression levels of the peptidase ScpC (also called SpyCEP), but the SLS protoxin *sagA* (discussed above) and the virulence-associated ABC transporter component *siaA* were upregulated in response to SilCR. Furthermore, rather than protecting the mice from infection-associated pathology, treatment with SilCR resulted in a delay in wound healing (Salim et al., 2008).

However, other studies have indicated that the SilCR pheromone may actually protect the host from invasive Streptococcal disease. The addition of exogenous SilCR protected mice from GGS-mediated mortality and reduced bacterial dissemination in a mouse model of infection, indicating that the SilCR pheromone actually suppresses pathogenicity (Michael-Gayego et al., 2013). This hypothesis is supported by other studies indicating that the addition of SilCR reduces the ability of GAS to avoid immune detection by preventing GAS from inducing the proteolytic cleavage of the pro-inflammatory cytokine IL-8 (Hidalgo-Grass et al., 2004; Eran et al., 2007). This activity was shown to be related to the production of ScpC, which has since been well-characterized as an important virulence factor that is regulated by the SilCR and is important for enabling innate immune evasion (Edwards et al., 2005; Hidalgo-Grass et al., 2006; Sumby et al., 2008; Kurupati et al., 2010; Chiappini et al., 2012). Interestingly, this is in direct contrast to results discussed previously (Salim et al., 2008), indicating potential strain or context-dependent effects of this operon. Based on these results, it appears that the *sil* locus may actually be advantageous for Streptococcal pathogenesis in the absence of the pheromone, and highlights the importance of strains that have the *sil* locus but are unable to produce the SilCR pheromone with regards to their propensity for causing severe disease.

## 4 Discussion and conclusions

Streptococci produce many factors that enable them to interact with their hosts for their benefit. These factors can help the bacteria colonize and establish a foothold in their host, compete with other microbes in their niche, and cause and progress disease, among other roles. Small peptides have become increasingly appreciated as Streptococcal factors that contribute to these processes, and can have significant effects on host-microbe biology despite their relatively small size. An overview of these peptides and their functions is shown in **Figure 1**. Some peptides, such as the virulence factor Streptolysin S, directly interact with host cells to induce pro-inflammatory signaling cascades and cytotoxicity, allowing the producing *Streptococcus* to increase disease severity during infections. Others, such as the short hydrophobic peptides (SHPs) that bind cytosolic regulator of glucosyltransferase gene (Rgg) transcription factors and the double-glycine containing peptides that are recognized by membrane-bound sensor kinases, shape Streptococcal interactions with their hosts by regulating the expression of a variety of genes, including those associated with colonization, virulence, and competence. Altogether, it is apparent that peptides are an important part of the diverse arsenal that is utilized by Streptococcal species under a multitude of conditions.

The impact of Streptococcal peptides on host immune responses is not limited to the traditional functions of the peptide on bacterial processes or virulence. Recent evidence is increasingly demonstrating that host cells are able to directly sense bacterial products that are associated with inter-bacterial signaling, thereby allowing them to respond to potential pathogens. QS-associated pheromones in Gram-negative bacteria, which are typically non-proteinaceous in nature, have been known to trigger immune responses in host cells (Verbeke et al., 2017; Xiao et al., 2022). Furthermore, QS-associated peptide pheromones from other Gram-positive bacteria are known to stimulate immune responses in host cells, and host GPCRs have often been associated with their detection. As described above, the quorum sensing-associated pheromone CSP-1 can be directly sensed by host cells through GPCRs and trigger an immune response (Pundir et al., 2019; Medapati et al., 2021; Nagi et al., 2023). One study screened 89 peptide pheromones from Gram-positive bacteria for their ability to induce the production of IL-6 and TNF-α in murine splenocytes and J774 macrophages. This screen identified several peptides that induced a significant immune response, including hits from *Staphylococcus aureus*, *Staphylococcus epidermidis*, *Bacillus cereus*, *Bacillus thuringiensis*, and *Enterococcus faecalis*. This study also identified the *Bacillus* peptide pheromone PapR71 in human plasma, indicating that bacterial signaling peptides with immunomodulatory activity may be biologically relevant in the host context (De Spiegeleer et al., 2023). The evaluation of bacterial peptides traditionally associated with quorum sensing for their ability to induce host immune responses therefore represents an interesting approach for identifying new ways by which bacteria modulate their host environment. It is also possible that some small peptides with similar activity may be awaiting discovery, indicating the importance of re-evaluating bacterial genomes to identify new factors that may be important for how hosts interact with Streptococcal species (Kaspar and Walker, 2019).

Evidence that peptides produced by *Streptococcus* species can modulate host immunity and contribute to pathogenesis in several ways suggests that disrupting the typical functions of these peptides holds promise for the development of therapeutics for treating Streptococcal infections. For example, several studies have explored ways to inhibit SLS-mediated effects on host cells. Small molecule inhibitors of proteins involved in ion transport, such as DIDS (4,4’-diisothiocyanatostilbene-2,2’-disulfonate) and the N-cyanosulfonamide S0859 have been used to inhibit the lysis of erythrocytes and keratinocytes in response to SLS (Higashi et al., 2016; Hammers et al., 2022). Interestingly, S0859 treatment was also able to prevent SLS-mediated NF-κB activation in keratinocytes, indicating that small molecule inhibition of the toxin can also prevent the associated damaging pro-inflammatory responses (Hammers et al., 2022). However, S0859 had no apparent effect when used to treat mice infected with GAS, while DIDS treatment reduced lesion sizes in mice exposed to SLS-producing GAS (Higashi et al., 2016; Hammers et al., 2022). Furthermore, inhibition of SLS-mediated IL-1β activity with a neutralizing antibody also reduced the size of skin lesions and wound-associated CFUs in mice when the antibody was co-administered subcutaneously with GAS (Flaherty et al., 2018). Together, these studies indicate that targeted inhibition of SLS binding with its protein targets and antagonism of the associated hyperinflammatory response to SLS have the potential to reduce the severe effects of SLS in invasive GAS infections. Quorum sensing inhibition has also been pursued as a strategy for treating bacterial infections. Multiple reviews have detailed the various approaches that have been used to disrupt bacterial communication pathways that contribute to virulence (Zhu and Lau, 2011; LaSarre and Federle, 2013; Rémy et al., 2018; Naga et al., 2023). Overall, there are three major approaches for disrupting QS: (i) inhibiting pheromone biosynthesis, (ii) targeting the pheromone receptors on responding cells, and (iii) targeted degradation of the pheromones themselves, although other approaches have been described. These strategies generally take an anti-virulence approach to treatment rather than bacteriostatic or bactericidal antimicrobial activity, and thereby represent potential alternatives to traditional antibiotics (Naga et al., 2023).

Small peptides are able to control several processes in streptococci, many of which are important for interacting with their hosts and enabling virulence. As a result, many of these peptides are associated with modulating host immunity, through both direct and indirect mechanisms. Although several recent advances have been made towards understanding how these peptides contribute to streptococci-host interactions, many additional questions remain unanswered. The discovery of new peptides and revisiting previously known peptides that may have immunomodulatory activity should continue to occur in the upcoming years, and the results of these studies have major implications for global health through treating Streptococcal infections.

## Author contributions

DH: Conceptualization, Funding acquisition, Writing – original draft, Writing – review & editing. EW: Writing – original draft, Writing – review & editing.
